# Supplementary material for: Understanding the mechanisms of infodemics: Equation-based vs. agent-based models
Source: PLoS One. 2025 Dec 17;20(12):e0338614. doi: 10.1371/journal.pone.0338614 (PMC12711016; doi:10.1371/journal.pone.0338614)
Supplement: Appendix I — List of the equation-based models and their parameters, together with the state transition representations. (PDF) [file pone.0338614.s001.pdf]

Supplementary Materials

Appendix I: Equation-based SEIRS-class Models

Table 1: SEIRS models notations for variables and paramaters

**Variable**	**Name**	**Variable**	**Name**
N	Total population	β	Infection rate
S	Susceptible population	σ	Incubation rate
E	Exposed population	γ	Recovery rate
I	Infected population	ξ	Immunity loss rate
R	Recovered population		

SI Model

$$\begin{cases} S'(t) = -\beta N^{-1} S(t) I(t) \\ I'(t) = \beta N^{-1} S(t) I(t) \end{cases} \tag{1}$$

SIS Model

$$\begin{cases} S'(t) = -\beta N^{-1} S(t) I(t) + \gamma I \\ I'(t) = \beta N^{-1} S(t) I(t) - \gamma I \end{cases} \tag{2}$$

SIR Model

$$\begin{cases} S'(t) = -\beta N^{-1} S(t) I(t) \\ I'(t) = \beta N^{-1} S(t) I(t) - \gamma I(t) \\ R'(t) = \gamma I(t) \end{cases} \tag{3}$$

SIRS Model

$$\begin{cases} S'(t) = -\beta N^{-1} S(t) I(t) + \xi R(t) \\ I'(t) = \beta N^{-1} S(t) I(t) - \gamma I(t) \\ R'(t) = \gamma I(t) - \xi R(t) \end{cases} \tag{4}$$

SEIR Model

$$\begin{cases} S'(t) = -\beta N^{-1} S(t) I(t) \\ E'(t) = \beta N^{-1} S(t) I(t) - \sigma E(t) \\ I'(t) = \sigma E(t) - \gamma I(t) \\ R'(t) = \gamma I(t) \end{cases} \tag{5}$$

SEIRS Model

$$\begin{cases} S'(t) = -\beta N^{-1} S(t) I(t) + \xi R(t) \\ E'(t) = \beta N^{-1} S(t) I(t) - \sigma E(t) \\ I'(t) = \sigma E(t) - \gamma I(t) \\ R'(t) = \gamma I(t) - \xi R(t) \end{cases} \tag{6}$$

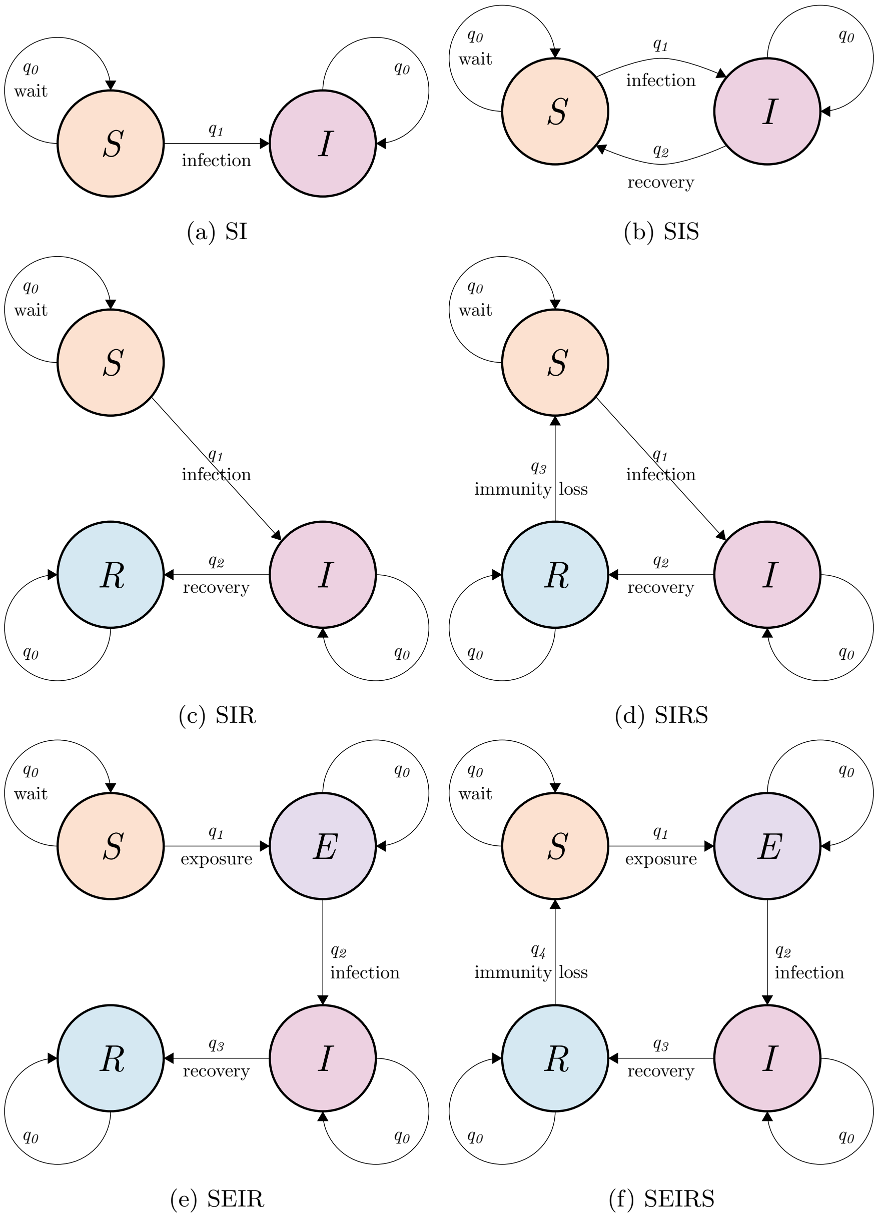

Figure 1: SI(S), SIR(S), and SEIR(S) model representation as finite-state machines in which population subsets mass-transition between states based on probabilities with states (S susceptible, E exposed, R recovered, I infected) and transitions (q_0 wait for a trigger in current state, q_1 exposure, q_2 infection, q_3 recovery, q_4 immunity loss).
