# Supplementary material for: Understanding the mechanisms of infodemics: Equation-based vs. agent-based models
Source: PLoS One. 2025 Dec 17;20(12):e0338614. doi: 10.1371/journal.pone.0338614 (PMC12711016; doi:10.1371/journal.pone.0338614)
Supplement: Appendix II — The flow diagram for model scheduling and the interfaces of the two ABMs. (PDF) [file pone.0338614.s002.pdf]

Appendix II: Implementation

Figure 2 shows the flow diagram for model scheduling. Figures 3 and 4 show the interfaces for the two models: simple ABM and enhanced ABM.

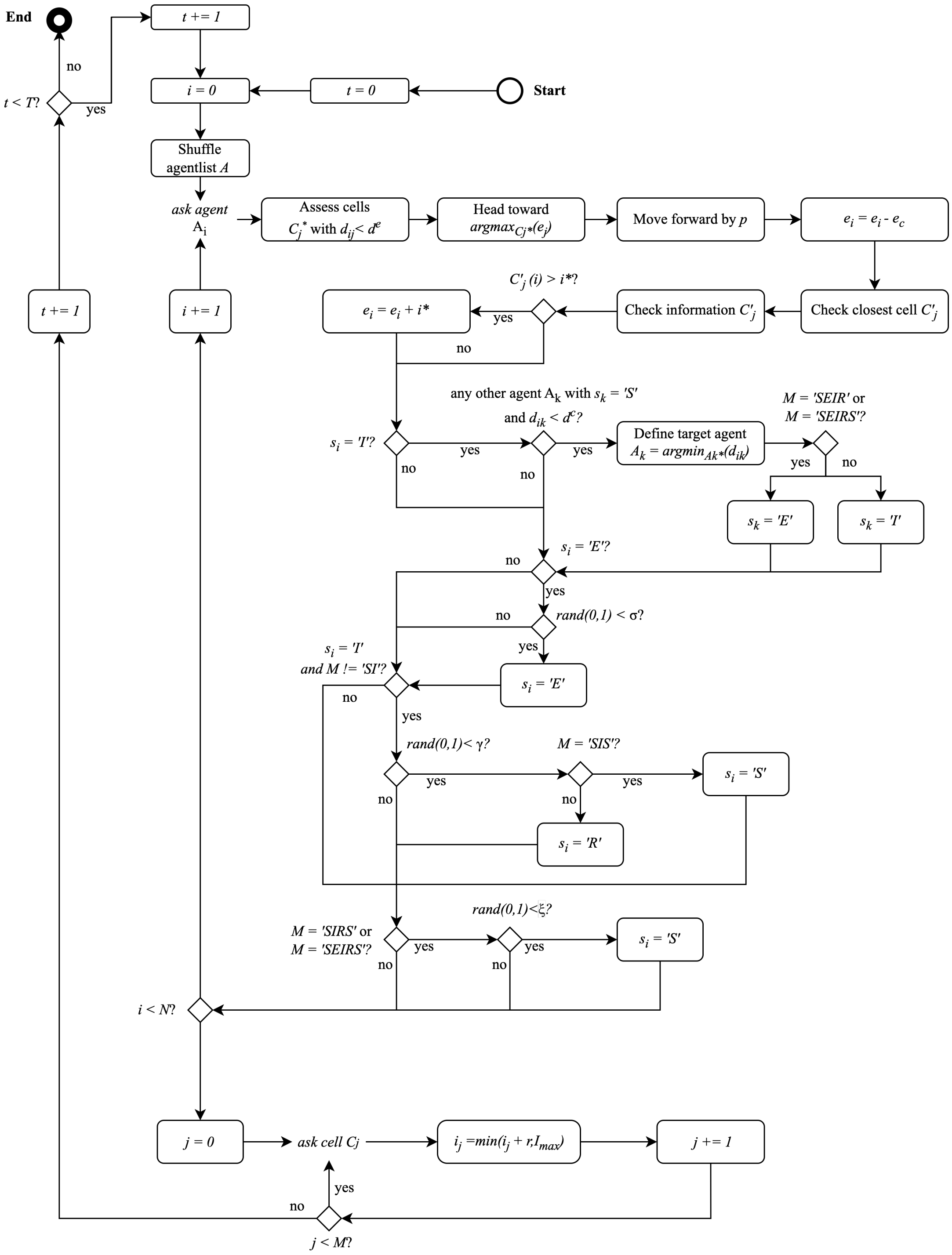

Figure 2: Flow diagram of the model scheduling

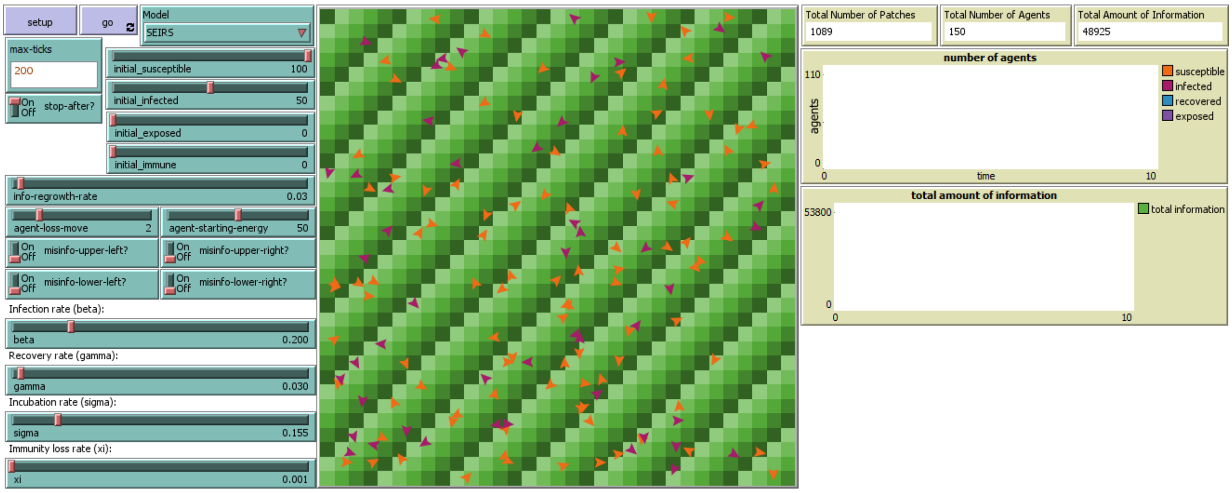

Figure 3: User interface for the simple ABM.

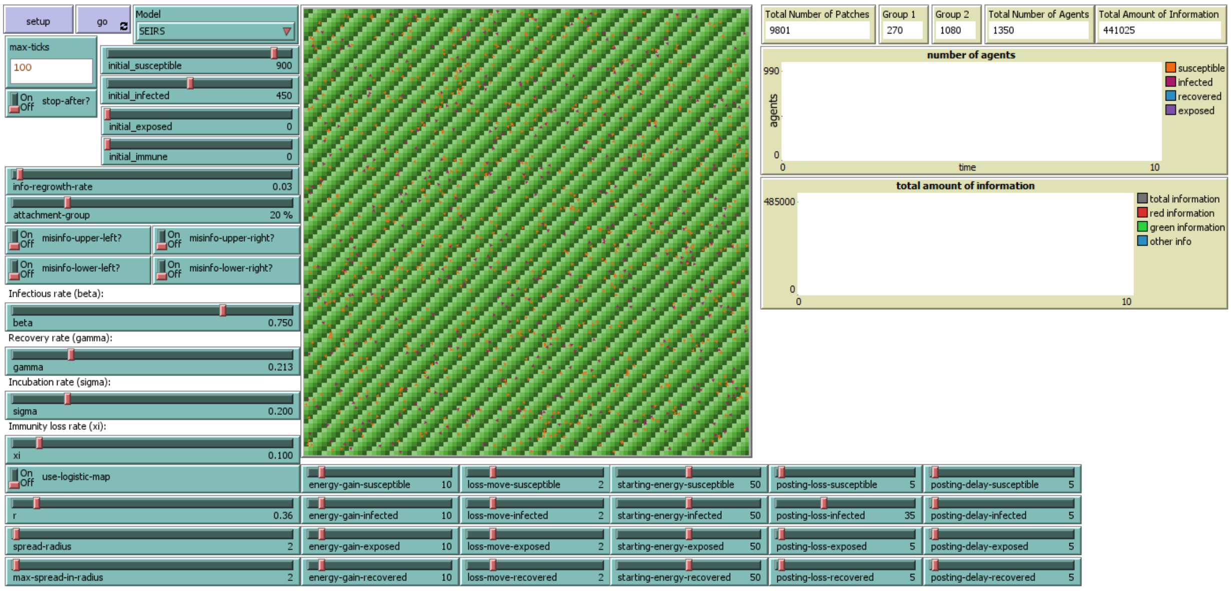

Figure 4: User interface for the enhanced ABM.
