# Supplementary material for: Understanding the mechanisms of infodemics: Equation-based vs. agent-based models
Source: PLoS One. 2025 Dec 17;20(12):e0338614. doi: 10.1371/journal.pone.0338614 (PMC12711016; doi:10.1371/journal.pone.0338614)
Supplement: Appendix III — Definitions and algorithms specific to the implementation fo the two ABMs. (PDF) [file pone.0338614.s003.pdf]

Appendix III: Algorithms

The following definitions and algorithms are specific to the implementation of the two ABMs using the NetLogo language, and are presented in pseudo-code form for readability.

Simple case

Let $\mathcal{A}$, the set of agents defined by:

$$\mathcal{A} = \{(x_i, y_i, h_i, s_i, e_i) | x_i, y_i \in [p_{min}, p_{max}], h_i \in [0, 360), s_i \in \{S, E, I, R\}, e_i \in [0, e_{max}]\}, \tag{7}$$

where x_i and y_i are the coordinates of the agent in the environment, h_i is the heading angle of the agent, and s_i is the infection state of the agent: S for susceptible, E for exposed, I for infected and R for recovered; e_i is the energy of the agent. The maximum amount of energy an agent ca have at any point in time is $e_{max} = 100$ for all simulations.

In addition to $\mathcal{A}$, we define subsets for each state value s_i so that:

$$\mathcal{A} = \mathcal{S} \cup \mathcal{E} \cup \mathcal{I} \cup \mathcal{R}, \tag{8}$$

where $\mathcal{S}$, $\mathcal{E}$, $\mathcal{I}$ and $\mathcal{R}$ are defined by:

$$\begin{cases} \mathcal{S} = \{a_i \in \mathcal{A} | \forall a_i = (x_i, y_i, h_i, s_i, e_i), s_i = S\} \\ \mathcal{E} = \{a_i \in \mathcal{A} | \forall a_i = (x_i, y_i, h_i, s_i, e_i), s_i = E\} \\ \mathcal{I} = \{a_i \in \mathcal{A} | \forall a_i = (x_i, y_i, h_i, s_i, e_i), s_i = I\} \\ \mathcal{R} = \{a_i \in \mathcal{A} | \forall a_i = (x_i, y_i, h_i, s_i, e_i), s_i = R\} \end{cases} \tag{9}$$

Let P, the set of patches (NetLogo-specific term for cell) defined by:

$$\mathcal{P} = \{(x_i, y_i, q_i) | x_i, y_i \in \mathbb{N}, q_i \in [0, 100]\} \tag{10}$$

where x_i and y_i are the coordinates of the patch, q_i is the total quantity of information on the patch, and

$$|\mathcal{P}| = (p_{max} - p_{min} + 1)^2. \tag{11}$$

Let $\mathcal{M}$ the set of models defined by:

$$\mathcal{M} = \{SI, SIS, SIR, SIRS, SEIR, SEIRS\} \tag{12}$$

Enhanced case

Let $\mathcal{A}^*$, the set of agents defined by:

$$\mathcal{A}^* = \{(x_i, y_i, h_i, s_i, e_i, g_i) | x_i, y_i \in [p_{min}, p_{max}], h_i \in [0, 360), s_i \in \{S, E, I, R\}, e_i \in [0, e_{max}], g_i \in \{G1, G2\}\}, \tag{13}$$

where x_i and y_i are the coordinates of the agent in the environment, h_i is the heading angle of the agent, and s_i is the infection state of the agent: S for susceptible, E for exposed, I for infected and R for recovered; g_i is the group of the agent, and e_i is the energy of the agent. The maximum amount of energy an agent can have at any point in time is $e_{max} = 100$ for all simulations.

In addition to $\mathcal{A}^*$, we define subsets for each state value s_i so that:

$$\mathcal{A}^* = \mathcal{S}^* \cup \mathcal{E}^* \cup \mathcal{I}^* \cup \mathcal{R}^*, \tag{14}$$

where $\mathcal{S}^*$, $\mathcal{E}^*$, $\mathcal{I}^*$ and $\mathcal{R}^*$ are defined by:

$$\begin{cases} \mathcal{S}^* = \{a_i \in \mathcal{A}^* | \forall a_i = (x_i, y_i, h_i, s_i, e_i, g_i), s_i = S\} \\ \mathcal{E}^* = \{a_i \in \mathcal{A}^* | \forall a_i = (x_i, y_i, h_i, s_i, e_i, g_i), s_i = E\} \\ \mathcal{I}^* = \{a_i \in \mathcal{A}^* | \forall a_i = (x_i, y_i, h_i, s_i, e_i, g_i), s_i = I\} \\ \mathcal{R}^* = \{a_i \in \mathcal{A}^* | \forall a_i = (x_i, y_i, h_i, s_i, e_i, g_i), s_i = R\} \end{cases} \tag{15}$$

Let P, the set of patches defined by:

$$\mathcal{P}^* = \{(x_i, y_i, q_i, c_i) | x_i, y_i \in \mathbb{N}, q_i \in [0, 100], c_i \in \{green, red\}\} \tag{16}$$

where x_i and y_i are the coordinates of the patch, q_i is the total quantity of information on the patch, and

$$|\mathcal{P}^*| = (p_{max} - p_{min} + 1)^2. \tag{17}$$

Let $\mathcal{M}$ the set of models defined by:

$$\mathcal{M} = \{SI, SIS, SIR, SIRS, SEIR, SEIRS\} \tag{18}$$

Functions

The NetLogo functions are used by the algorithms in this section:

in-cone (agent, patch set, radius, viewing angle) This function returns all the patches, a subset of patch set, that are part of a vision distance (radius) and the viewing angle of the agent;

in-radius (agent, patch set, radius) This function returns all the patches, a subset of patch set, that are part of a vision distance (radius) of the agent;

forward (agent) This function moves the agent forward in the agent heading direction and returns the new coordinates (x, y) for the agent;

closest-susceptible (agent) This function returns the closest (minimum distance) susceptible agent from current agent;

min-n-of (agent, patch set, max agents, spread radius) This function returns a list of agents with $maximumsize = maxagents$, containing closest agents in $radius = spreadradius$ from current agent. The returned list of agents contains all agents regardless of the type (susceptible, infected, exposed or recovered);

patch-here (agent) This function returns the patch (cell) where the agent is located;

energy-gain (agent state) This function return the energy gain from consuming information for a given agent state (S, E, I or R). The energy gain for each state is defined in the NetLogo ABM user interface;

posting-delay (agent state) This function returns true if the posting delay (number of simulation ticks) passed for a given agent state (S, E, I or R), or false otherwise. The posting delay for each state is defined in the NetLogo ABM user interface;

posting-loss (agent state) This function return the energy loss from posting information for a given agent state (S, E, I or R). The posting loss for each state is defined in the NetLogo ABM user interface.

Algorithms for behaviors

Algorithms 1-9 show the state transitions of agents (i.e., agent behaviors) for the simple and enhanced models, for all six types: *SI, SIS, SIR, SIRS, SEIR, SEIRS*. All algorithms for the simple case are using the $\mathcal{A}$ and $\mathcal{P}$ sets while all algorithms for the enhanced case are using the $\mathcal{A}^*$ and $\mathcal{P}^*$ sets.

Algorithm 1 defines the movement behavior of the agents for the Simple and Enhanced cases, and applies to all agents in the ABM (line 1). The agents are moving toward the patch (cell) with the highest amount of information from their neighbourhood (lines 2-3). For all simulations we considered the agent $radius = 4$ and viewing $angle = 60$ degrees. If the agent cannot find a patch using the in-cone() function because the agent reached a corner, in-radius function is used instead (lines 4-8). N.B.: All simulations were done using torus environment, therefore only in-cone() function was used. We included in-radius() function in this algorithm for compatibility reasons with box environments. After the agent heading is set (lines 5 and 7), the agent moves forward toward the heading (line 8).

Algorithm 2 defines the misinformation spread behavior of agents for the Simple case. For all infected agents in the ABM (lines 1-3), we check which is the closest agent with state S (susceptible) and we call the susceptible agent a_j (line 4). If the condition random(1000) $< \beta \cdot 1000 \cdot 2 \cdot \frac{|\mathcal{P}|}{|\mathcal{S}|}$ is met, the infected agent a_i infects the susceptible agent a_j (lines 5-12). If the model is SEIR or SEIRS, agent a_j first becomes exposed before becoming infected (lines 6-8).

Algorithm 3 defines the incubation behavior of agents for the Simple and Enhanced cases. This behavior applies only for SEIR and SEIRS models. For all exposed agents (lines 1-3), if the random(1000) $< \sigma \cdot 1000$ condition is met (line 4), then the agent becomes infected (line 5).

Algorithm 4 defines the healing behavior of the infected agents for the Simple and Enhanced cases. This behavior applies to all models except SI. For each infected agent (lines 1-3), if the model is SIS (line 4) then if the random(1000) $< \gamma \cdot 1000$ condition is met (line 6), the agent becomes susceptible (line 6). If the model is one of: SIR, SIRS, SEIR or SEIRS (line 8), then if the random(1000) $< \gamma \cdot 1000$ condition is met, then the agent becomes recovered (line 10).

Algorithm 5 defines the immunity loss behavior for the Simple and Enhanced cases. This behavior applies only to the SIRS and SEIRS models. For each recovered (immune) agent (lines 1-3), if the model is SIRS or SEIRS (line 4) and the random(1000) $< \xi \cdot 1000$ condition is met, then the agent becomes susceptible (line 6).

Algorithm 6 defines the misinformation spread behavior for the Enhanced case. For each infected agent (lines 1-3), we check the number susceptible agents in $radius = spread\text{-}radius$ (line 4), where *spread-radius* is a parameter defined

in the NetLogo ABM user interface. If the number of susceptible agents in radius is greater than *max-spread-in-radius*, we keep only the closest agents in the list. *max-spread-in-radius* is also a parameter defined in the NetLogo ABM user interface. After finding the set of closest susceptible agents in this radius, we check if the random(1000) $< \beta \cdot 1000 \cdot \frac{|\mathcal{I}^*|}{|\mathcal{A}^*|}$ condition is met (line 5). If the condition is met, then if the model is SEIR or SEIRS (line 6), all susceptible agents in the radius with the same group become exposed (lines 8-12), and if the model is different, then all the susceptible agents in radius with the same group become infected (lines 15-19). Point-to-point direct misinformation spread from infected agents to susceptible agents is possible only if the agents are part of the same group (lines 9 and 16). However, a susceptible agent can be infected indirectly by an infected agent form a different group through the posting and consumption mechanism. Please note that this algorithm does not include the misinformation spread caused by consuming false information from the environment. Instead, information posting and consumption behaviors for the enhanced case are defined by algorithms 7 and 8.

Algorithm 7 defines the information consumption behavior for the Enhanced case. For each agent we check the color of the patch (cell) where the agent is located (lines 1-3). If the color is green and the quantity of information on the patch is greater than or equal to 10 (lines 3-9), then the agent consumes the information from the patch and the energy of the agent is increased by the same amount (lines 7-8). If the color is red and the quantity of information on the patch is greater than or equal to 10 (lines 10-17), then the agent consumes the information from the patch. the energy of the agent is increased by the same amount (lines 13-14), and the agent becomes infected (line 15).

Algorithm 8 defines the information posting behavior for the Enhanced case. For each agent, if the number of simulation ticks divides the amount returned by the posting-delay() function and the energy of the agent is greater than 90, then the agent creates a post (lines 1-2). The posting delay is defined in the NetLogo ABM user interface for each agent state (S, E, I and R) and it is constant throughout the simulation. When an agent creates a post (lines 3-17), the post is created on the same patch (cell) where the agent is located (line 4), and the amount of information on this patch is increased by *info-regrow-rate* $\cdot 100$ (line 5), where *info-regrow-rate* is a parameter defined in the NetLogo ABM user interface. When the agent creates a post, the agent loses the amount of energy returned by the posting-loss() function (line 10). The posting loss is defined in the NetLogo ABM user interface for each agent state (S, E, I and R) and it is constant throughout the simulation. If the agent is infected (line 11), then the patch (cell) color becomes red (line 13), otherwise it becomes green (line 15).

Algorithm 9 defines the information growth behavior for the Enhanced case. The green information on the patch is growing by *info-regrow-rate* $\cdot 100$ (line 6), the information on the red patches is not growing, and a red patch becomes green when red information drops below 10 (lines 13-16).

Algorithm 1: Agent movement behavior (Simple and Enhanced cases)

Data: $a_i \in \mathcal{A}, p_i \in \mathcal{P}, model \in \mathcal{M}, \beta, \gamma, \sigma, \xi$

for $a_i \in A$ **do**
    target $\leftarrow$ max(in-cone($a_i, \mathcal{P}, 4, 60$)) // in-cone() function returns all the patches, a subset of $\mathcal{P}$, that are part of a vision distance (radius) and the viewing angle of the agent a_i. In this case the radius is 4 and the viewing angle is 60 degrees. The *target* variable becomes the patch from the subset with the highest amount of information available.
    target-backup $\leftarrow$ max(in-radius($a_i, \mathcal{P}, 4$)) // *target-backup* is used only when the agent reaches a corner and in-cone() no longer returns a valid target. The in-radius() function behaves similarly as the in-cone() function, with the exception that it considers the viewing angle of the agent a_i = 360 degrees.
    if $target \neq nobody \wedge target \neq patch\text{-}here$ **then**
        $h_i \leftarrow target$ // agent a_i's heading attribute becomes *target*
    else
        $h_i \leftarrow target\text{-}backup$ // agent a_i's heading attribute becomes *target-backup*
    end
    $(x_i, y_i) \leftarrow$ forward(a_i) // agent a_i moves forward
end

Algorithm 2: Agent misinformation spread behavior (Simple case)

Data: $a_i \in \mathcal{A}, p_i \in \mathcal{P}, model \in \mathcal{M}, \beta, \gamma, \sigma, \xi$

for $a_i \in A$ **do**
  if $s_i = I$ // if agent a_i is infected
  then
    $a_j \leftarrow$ closest-susceptible(a_i) // a_j is initialized as the closest susceptible agent (minimum distance) from agent a_i
    if random$(1000) < \beta \cdot 1000 \cdot 2 \cdot \frac{|\mathcal{P}|}{|\mathcal{S}|}$ **then**
      if $model = SEIR \lor model = SEIRS$ // If the model includes exposed population
      then
        $s_j \leftarrow E$ // agent a_j becomes exposed
      else
        $s_j \leftarrow I$ // agent a_j becomes infected
      end
    end
  end
end

Algorithm 3: Exposed agent becomes infected (Simple and Enhanced cases, only for SEIR and SEIRS)

Data: $a_i \in \mathcal{A}, p_i \in \mathcal{P}, model \in \mathcal{M}, \beta, \gamma, \sigma, \xi$

for $a_i \in A$ **do**
  if $s_i = E$ // agent a_i is exposed
  then
    if random$(1000) < \sigma \cdot 1000$ **then**
      $s_i \leftarrow I$ // agent a_i becomes infected
    end
  end
end

Algorithm 4: Infected agent healing behavior (Simple and Enhanced cases, for all models except SI)

Data: $a_i \in \mathcal{A}, p_i \in \mathcal{P}, model \in \mathcal{M}, \beta, \gamma, \sigma, \xi$

for $a_i \in A$ **do**
  if $s_i = I$ // if agent a_i is infected
  then
    if $model = SIS$ **then**
      if random$(1000) < \gamma \cdot 1000$ **then**
        $s_i \leftarrow S$ // agent a_i becomes susceptible
      end
    else if $model \in \{SIR, SIRS, SEIR, SEIRS\}$ **then**
      if random$(1000) < \gamma \cdot 1000$ **then**
        $s_i \leftarrow R$ // agent a_i becomes recovered (immune)
      end
    else
      // do nothing when the model is SI
    end
  end
end

Algorithm 5: Agent immunity loss behavior (Simple and Enhanced cases, only for SIRS and SEIRS)

Data: $a_i \in \mathcal{A}, p_i \in \mathcal{P}, model \in \mathcal{M}, \beta, \gamma, \sigma, \xi$

for $a_i \in A$ **do**
if $s_i = R$ // if agent a_i is recovered (immune)
then
if $model \in \{SIRS, SEIRS\}$ **then**
if random(1000) $< \xi \cdot 1000$ **then**
$s_i \leftarrow S$ // agent a_i becomes susceptible
end
end
end
end

Algorithm 6: Agent misinformation spread behavior (Enhanced case)

Data: $a_i \in \mathcal{A}^*, p_i \in \mathcal{P}^*, model \in \mathcal{M}, \beta, \gamma, \sigma, \xi$

for $a_i \in A^*$ **do**
if $s_i = I$ // if agent a_i is infected
then
$\mathcal{A}^*_{in\text{-}radius}$ = min-n-of($a_i, \mathcal{P}^*$, *max-spread-in-radius*, *spread-radius*) // the min-n-of() function returns the list of closest agents in $radius = spread\text{-}radius$. The maximum number of elements in the list is *max-spread-in-radius*.
if random(1000) $< \beta \cdot 1000 \cdot \frac{|\mathcal{I}^*|}{|\mathcal{A}^*|}$ **then**
if $model = SEIR \vee model = SEIRS$ // If the model includes exposed population
then
for $a_j \in \mathcal{A}^*_{in\text{-}radius}$ **do**
if $g_i = g_j$ // check if agent a_i and agent a_j are part of the same group
then
$s_j \leftarrow E$ // agent a_j becomes exposed
end
end
else
for $a_j \in \mathcal{A}^*_{in\text{-}radius}$ **do**
if $g_i = g_j$ // check if agent a_i and agent a_j are part of the same group
then
$s_j \leftarrow I$ // agent a_j becomes infected
end
end
end
end
end
end

Algorithm 7: Agent information consumption (Enhanced case)

Data: $a_i \in \mathcal{A}^*, p_i \in \mathcal{P}^*, model \in \mathcal{M}, \beta, \gamma, \sigma, \xi$
for $a_i \in A^*$ **do**
  $p_j \leftarrow \text{patch-here}(a_i)$ // initialize patch (cell) p_j as the patch where agent a_i is located
  if $c_j = green$ // if current patch (cell) p_j has true information (is green)
  then
    if $q_j >= 10$ // if the quantity of information on current patch (cell) p_j is greater than or equal with 10
    then
      $e_i \leftarrow e_i + \text{energy-gain}(s_i)$ // increase the energy of agent a_i by the amount returned by function energy-gain()
      $q_j \leftarrow q_j - \text{energy-gain}(s_i)$ // decrease the quantity of information of patch (cell) p_i by the amount returned by function energy-gain()
    end
  else
    if $q_j >= 10$ // if the quantity of information on current patch (cell) p_j is greater than or equal with 10
    then
      $e_i \leftarrow e_i + \text{energy-gain}(s_i)$ // increase the energy of agent a_i by the amount returned by function energy-gain()
      $q_j \leftarrow q_j - \text{energy-gain}(s_i)$ // decrease the quantity of information of patch (cell) p_i by the amount returned by function energy-gain()
      $s_i \leftarrow I$ // agent a_i becomes infected
    end
  end
end

Algorithm 8: Agent information posting (Enhanced case)

Data: $a_i \in \mathcal{A}^*, p_i \in \mathcal{P}^*, model \in \mathcal{M}, \beta, \gamma, \sigma, \xi$
for $a_i \in A^*$ **do**
  if $\text{modulo}(ticks, \text{posting-delay}(s_i)) = 0 \land e_i > 90$ // agents post after every posting-delay(s_i) number of ticks if the energy of a_i is greater than 90
  then
    $p_j \leftarrow \text{patch-here}(a_i)$ // initialize patch (cell) p_j as the patch where agent a_i is located
    $q_j \leftarrow q_j + \textit{info-regrow-rate} \cdot 100$ // the quantity of information on patch (cell) p_j is increased by *info-regrow-rate* when the agent posts information
    if $q_j > 100$ // check if q_j exceeds the maximum amount of information quantity for patches (cells)
    then
      $q_j \leftarrow 100$ // set the quantity of information for patch (cell) p_j as the maximum amount of quantity for patches (cells)
    end
    $e_i \leftarrow e_i - \text{posting-loss}(s_i)$ // decrease the energy of agent a_i by the amount returned by the posting-loss() function
    if $s_i = I$ // if the agent is infected
    then
      $c_j \leftarrow red$ // the patch color becomes red (contains false information)
    else
      $c_j \leftarrow green$ // the patch color becomes green (contains true information)
    end
  end
end

Algorithm 9: Information growth algorithm (Enhanced case)

Data: $a_i \in \mathcal{A}^*, p_i \in \mathcal{P}^*, model \in \mathcal{M}, \beta, \gamma, \sigma, \xi$

for $p_i \in P^*$ **do**
    if $c_i = green$ // if current patch (cell) p_j has true information (is green)
    then
        if $q_i < 100$ // check if q_i (total quantity of information on patch (cell) p_i) is less than the maximum amount of information quantity for patches (cells)
        then
            $q_i \leftarrow q_i + \textit{info-regrow-rate} \cdot 100$ // the quantity of information on patch (cell) p_j is increased by $\textit{info-regrow-rate} \cdot 100$
            if $q_i > 100$ // check if q_j exceeds the maximum amount of information quantity for patches (cells)
            then
                $q_i \leftarrow 100$ // set the quantity of information for patch (cell) p_j as the maximum amount of quantity for patches (cells)
            end
        end
    end
    if $c_i = red \wedge q_i <= 10$ // if current patch (cell) p_i has false information (is red) and the total amount of information on the patch (cell) is less than or equal to 10
    then
        $c_i \leftarrow green$ // patch (cell) color becomes red
    end
end
