# Supplementary material for: Understanding the mechanisms of infodemics: Equation-based vs. agent-based models
Source: PLoS One. 2025 Dec 17;20(12):e0338614. doi: 10.1371/journal.pone.0338614 (PMC12711016; doi:10.1371/journal.pone.0338614)
Supplement: Appendix IV — Parameters and visualization of matching outcomes examples for the six SEIRS-class models. (PDF) [file pone.0338614.s004.pdf]

Appendix IV: Examples of matching outcomes

This section presents examples of matching outcomes with parameter values for β, γ, σ and ξ presented in Table 2. In the comparative visualization of time responses (Figures 5 to 10), we considered the average populations for the ABMs and the calculated populations for the EBMs. All results in this section are generated using the Simple ABM (presented in the paper) and the EBMs defined in Appendix I. The ABM responses show both the means for each population state over time for 1000 simulations, as well as the deviation tube (minimum and maximum).

Table 2: Preliminary results: matching outcomes between the ABM and EBMs based on the normalized mean root of square error NRMSE and the Pearson correlation coefficient ρ

Model	β	γ	σ	ξ	NRMSE				Pearson Coeff. ρ			
					S	E	I	R	S	E	I	R
SI	0.2	-	-	-	0.017	-	0.017	-	0.99	-	0.99	-
SIS	0.2	0.3	-	-	0.025	-	0.025	-	0.99	-	0.99	-
SIR	0.2	0.3	-	-	0.020	-	0.012	0.013	0.99	-	0.99	0.99
SIRS	0.2	0.3	-	0.001	0.021	-	0.012	0.013	0.99	-	0.99	0.99
SEIR	0.2	0.3	0.155	-	0.018	0.010	0.006	0.007	0.99	0.99	0.99	0.99
SEIRS	0.2	0.3	0.155	0.001	0.019	0.009	0.005	0.008	0.99	0.99	0.99	0.99

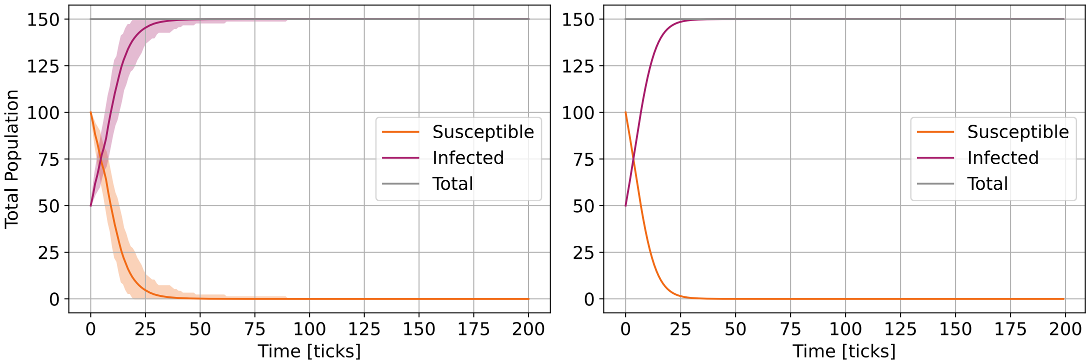

Figure 5: SI model comparison: ABM (left) and EBM (right) time responses.

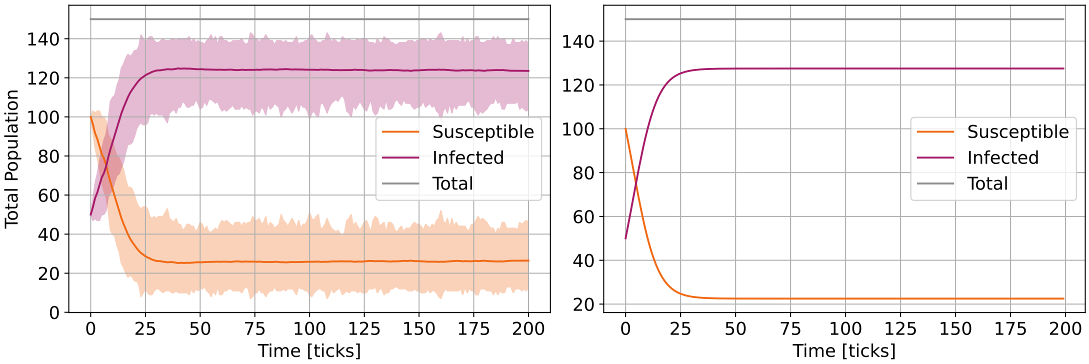

Figure 6: SIS model comparison: ABM (left) and EBM (right) time responses.

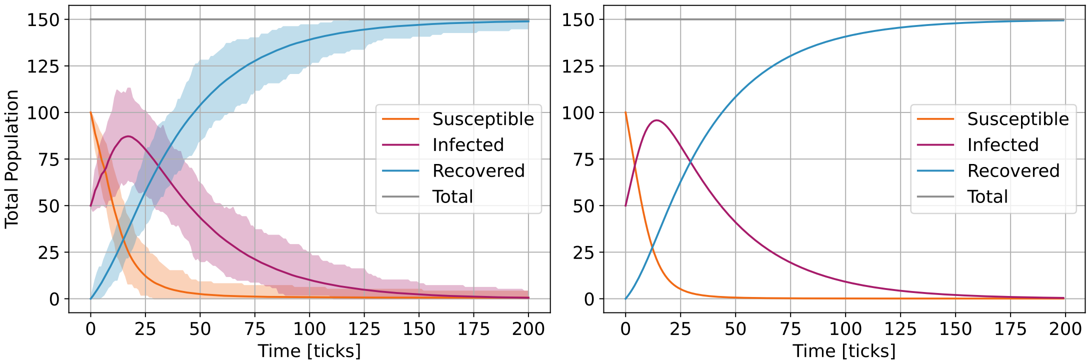

Figure 7: SIR model comparison: ABM (left) and EBM (right) time responses.

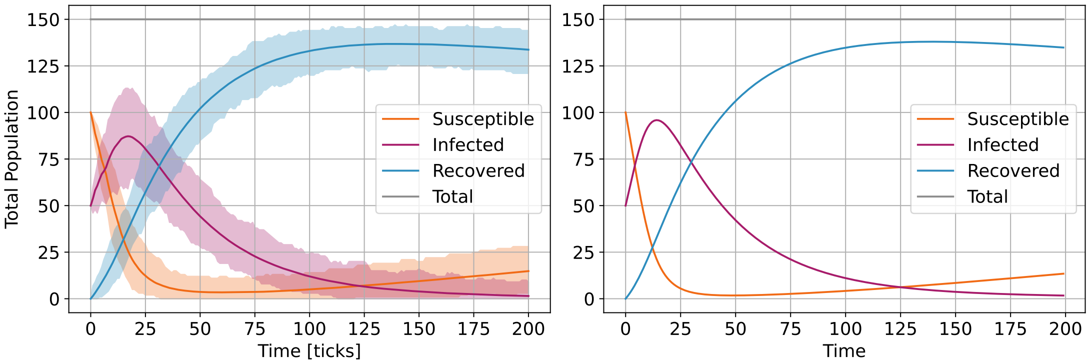

Figure 8: SIRS : ABM (left) and EBM (right) time responses.

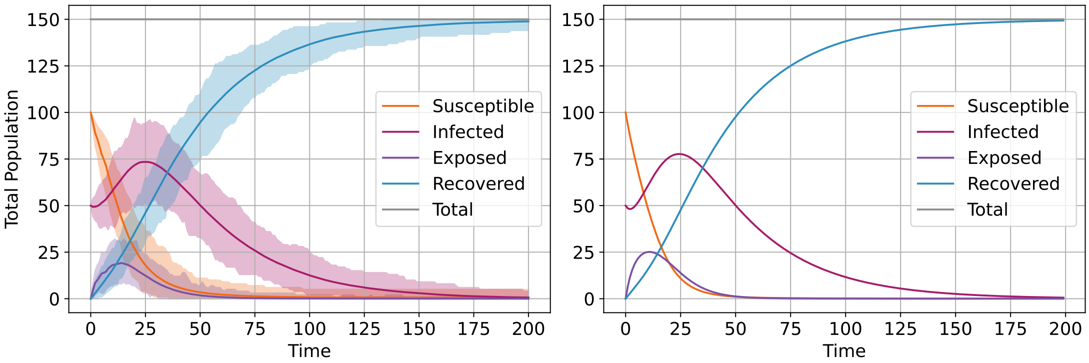

Figure 9: SEIR model comparison: ABM (left) and EBM (right) time responses.

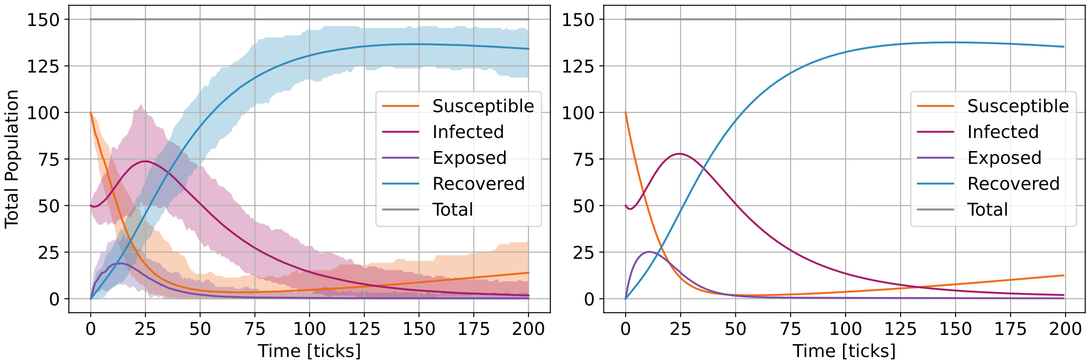

Figure 10: SEIRS model comparison: ABM (left) and EBM (right) time responses.
