# Supplementary material for: Understanding the mechanisms of infodemics: Equation-based vs. agent-based models
Source: PLoS One. 2025 Dec 17;20(12):e0338614. doi: 10.1371/journal.pone.0338614 (PMC12711016; doi:10.1371/journal.pone.0338614)
Supplement: Appendix V — Examples for the effect of homophily. (PDF) [file pone.0338614.s005.pdf]

Appendix V: Selected results (exemplification)

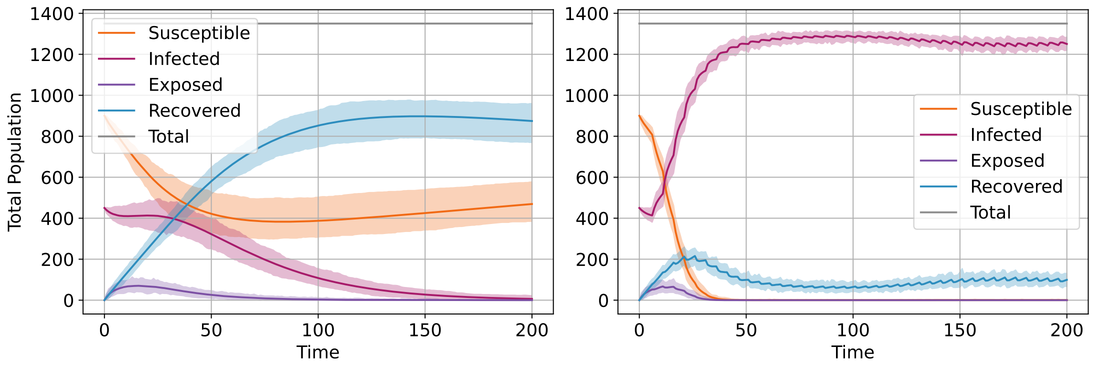

Figure 11: Effect of homophily $N_{G1} = 100\%$, $N_{G2} = 0\%$ (total $N = 1350$ agents): simple model (left) and enhanced model (right) for 1000 runs.

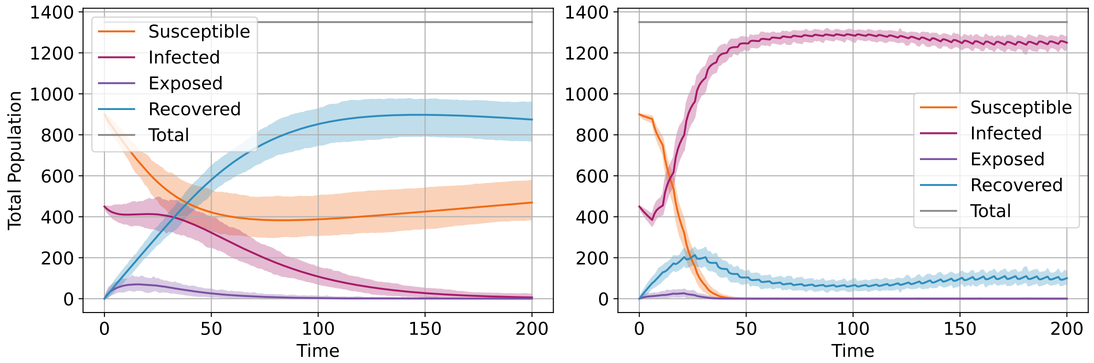

Figure 12: Effect of homophily $N_{G1} = 50\%$, $N_{G2} = 50\%$ (total $N = 1350$ agents): simple model (left) and enhanced model (right) for 1000 runs.
