# Supplementary material for: Understanding the mechanisms of infodemics: Equation-based vs. agent-based models
Source: PLoS One. 2025 Dec 17;20(12):e0338614. doi: 10.1371/journal.pone.0338614 (PMC12711016; doi:10.1371/journal.pone.0338614)
Supplement: Attachment — Submitted filename: response to reviewers.pdf [file pone.0338614.s007.pdf]

Response to Reviewers

June 2025

Dear editors and reviewers,

Thank you for the opportunity to revise our manuscript, and for the thorough comments. We have revised the paper in light of these useful suggestions. Our responses and changes to the manuscript can be found below (point-by-point for each item) and are as well highlighted in the uploaded revised manuscript.

1 Editor

In addition to the comments of the reviewers, I wonder if you have considered the related work in epidemics on networks, for example the excellent book by Miller and Kiss. The reason I mention this is that there is a spectrum from well-mixed ODE systems through to individually structured populations which can be represented on a network.

Thank you for your comment. We are familiar with the book, which we consider an excellent reference in the field. The aim of our work was to explore whether it is possible to represent the same diffusion phenomenon using models with fundamentally different structures but identical parameters. A possible direction (that we have investigated) would have been to include a network-based model as an intermediate step between a fully mixed ODE system and an agent-based approach. This still remains in our field of interest for further research, and at page 4 there is a review of these elements.

There are well-known results about how the dynamics change with the introduction of this kind of population structure (essentially, the epidemic will proceed more slowly and there can be depletion of susceptible individuals and in some cases patterns not unlike what you find in the continuous case with reaction-diffusion systems can arise).The cell-based ABM formulation is a kind of middle ground where interactions are locally well-mixed but with different localities (cells, in the ABM) but with transport between the cells. It would be interesting to see the work better situated in this landscape of modelling techniques.

Thank you for the suggestion. We have revised the text and included additional references to clarify the context, as you advised. In particular, we now

explicitly mention key works in the field, such as the seminal paper by Newman (2002), the review by Keeling and Eames (2005), and the comprehensive overview by Duan et al. (2015). This should make our approach and its position within the broader landscape of modeling techniques more transparent.

2 Reviewer 1

The background is generally well-written but could benefit from improved structure. Divide the section into subsections for better organization (e.g., "Diffusion Models Overview," "Epidemic Models," "Information Spread Models").

Thank you for your positive opinion and constructive feedback. We appreciate and had implemented your suggestion to better divide the background and the rest of the paper, for a greater readability.

Can use bullet points or tables to summarize and compare Equation-Based Models (EBMs) and Agent-Based Models (ABMs) for clarity.

Thank you for the suggestion, we appreciate the input. At the end of the models description section, there is now a summary table outlining the differences between EBM, simple ABM, and enhanced ABM. (Table 1)

The motivations and aims of the paper are clear. However better structuring (e.g., bullet points for the key objectives of EBM and ABM comparisons) would enhance clarity and focus.

Thank you for pointing this out. We restructured the aims and listed the key objectives under their two categories. (lines 48-64) (view Page 3)

The connection between the discussion on psychological perspectives and the modeling process is unclear. Clearly state how the points raised in the psychological perspectives section are incorporated into the model design. For instance, explicitly link psychological and psycho-social components to specific parameters, mechanisms, or agent behaviors.

Thank you for this comment. We added an explanatory note at the end of the psychological perspectives section. We hope this clarifies how we made our design choices, as we wanted to avoid repeating some of the technical methodological choices we describe in the next sections, which follow directly from the psych analysis. (view page 6 in section "Preliminary Analysis: a Psychological Perspective on the Infodemic Infection Mechanisms")

The discussion of (any) stochasticity in the ABM model is insufficient. Clarify how stochasticity is implemented (e.g., specific features of the NetLogo platform). Provide examples of how randomness may affect the results.

Thank you for catching this. We revised the section describing the inter-model comparison and clarified how we handle stochasticity across experiments (combined with the comment on fairness of the comparison). We provide examples of how randomness can affect results in the Results section, figures 3 and

4, with their associated explanations in text, where we show different variations around the mean for multiple ABM runs. Further examples of this are included in Appendices IV and V (page 15 in Result: Evaluation of intermodel equivalence).

Some technical aspects of the implementation (e.g., the use of energy) may overwhelm readers and can be moved into appendix.

Thank you for this comment. As energy is a state variable that we chose as a result of the psychological perspective analysis, we believe it should be described together with the other state variables. We have now linked this variable with the explanation on how the psych perspective has informed our design choices. (view Appendix II)

Comparative Analysis (ABM vs. EBM). The section lacks context (why is embedded there), and the motivations for performing the comparative analysis are unclear. Explain why this comparison is important and how it informs the rest of the paper. Clearly state how the findings from this section contribute to understanding the differences and complementarities between ABMs and EBMs.

We agree that the rationale for directly comparing ABMs and EBMs should be stated more clearly. We have revised the introduction of the section to clarify that this comparison is essential because EBMs are the traditional standard for modeling diffusion phenomena (especially when the number of entities in the system exceeds a threshold and make it hard to simulate on the computational side), but their underlying assumptions—such as population homogeneity and mean-field approximations—may overlook important individual-level mechanisms captured by ABMs. Systematically comparing the two approaches across a broad parameter range and fitting them to real-world data allows a better understand where they diverge and under what conditions each is more appropriate. So the findings from this section help clarify the specific strengths and limitations of both modeling paradigms, thereby informing the choice of method for future research on information diffusion and infodemics. Now, these elements are clearly highlighted in the section. (view page 12 in Comparative Analysis)

On Page 10, clarify what S_{RW} and I_{RW} represent.

We now specified these variables.

In Equation (4), explain the rationale for using the "min over k."

It was a typo, we meant min over π_i, which is the set of parameters over which the optimization has been performed. Thank you for noticing it, now it is corrected. (page 13 now corrected)

The term "preferential attachment" is used differently from its standard meaning in network science. Consider using a different term to avoid confusion. Clarify whether this mechanism is deterministic in your context.

Thank you for this point, we changed it to homophily, to illustrate a pref-

erence toward members of the same group. We clarified how the group sizes and assignments are specified in the simulations (combined with the comment regarding stochasticity).

To my understanding, the ABM has more parameters than the EBM, which could (inherently) make it easier to achieve a better fit to real data. Discuss this important potential limitation of the paper more explicitly.

Thank you for pointing out this important limitation. We have now added a dedicated section in the Discussion to address it . In brief, we clarify that the higher number of parameters in the enhanced ABM reflects its structural complexity, necessary to capture individual-level mechanisms and emergent dynamics, and that it is this better structural model adherence to the modelled system that explains the improved fit. (page 20 in Discussion)

On the same point, explore methods for a "fair" comparison between the two approaches, such as focusing on a small subset of key parameters in the ABM to identify those most critical for matching real data.

In this study we implemented the simple ABM as the fair comparison, as other functions associated with the complexity of human information exchanges are not included. To the same effect, the inter-model comparative analysis is reduced to the key parameters of the EBMs (e.g., infection rate, etc.), while the other ABM parameters are fixed - we now clarified this in the methodology. We also fixed the initialization of the ABM to reduce stochasticity, and also averaged the ABM experiments over several runs (standard practice in complex systems simulation). For the real world data, our goal was to assess the full power of each model, and so all parameters were utilized, with the same initialization of the map/seed; here, we included the time factor parameter to allow for a fair longitudinal sampling across models relative to the sampling of the real world data. The discussion can be found at page 18.

3 Reviewer 2

The paper "Understanding the mechanisms of infodemics: equation-based vs. agent-based models" presents a comparison of the two types of models (equation-vs agent-based) applied to the scenario of mis- and disinformation spreading, on the established analogy of epidemiologic modelling. The models are compared to each other on a wide range of variants (SI, SIS, etc.) and comprehensive parameter range, and also fitted to a real-world desinformation spread scenario. The conclusions present both the comparison of the models run, as well as the evaluation of the real-world data fitting. The code is well-written and organised and easy to check.

Thank you for your review. We truly appreciate the time and effort you dedicated to understanding the structure and aims of the paper, as well as you appreciation for the paper organization and the quality of the code.

On the whole the objective of the paper is valid and worth the publication, and the authors completed much of the necessary groundwork for the research. However, currently there are several points that need further work and clarification.

Thank you for acknowledging the relevance of our work and pointing out elements that could be improved to make it a better scientific output.

– The linguistic level of the presentation on the whole needs attention. Sentences like the below hinder the understanding and when revisited and improved, can add to the overall value of the paper. "Appendix II presents in the details the 359 models scheduling and their interfaces."

Thank you for pointing this out. We performed a language check throughout the paper and revised awkward sentences/typos.

– The framework in which the models are compared needs re-evaluation, as well as a clearer presentation.

Thank you for these critical comments. We revised the section describing the comparison and metrics (we answer the bullets points one by one below).

** The way the two models are compared and evaluated should be clarified. The paper uses two metrics (Pearson's correlation and normalized root mean square error), but neither of them are well motivated in the paper.*

Beside the elements described in the next bullet points, we added an explanation of why we chose the metrics and how we interpret them during the evaluation (pag. 13).

** Pearson's correlation coefficient only measures linear dependency, and it can have the value zero for dependent variables (if the dependence is not linear). It is not clear why we expect linear dependency: please, either clarify this or perhaps use Brownian (distance) covariance.*

Thank you for this comment, indeed the Pearson correlation measures linear dependency. But here we do not aim to check wether the two variables are dependent, as they result from two different models. What we test is the dependence of samples over time. This technique is often used in signal processing to check signal (or time series) similarity. To make a parallel with the statistical approach, we are checking the dependence between two outcome measures collected from two different populations/groups, cross-sectionally, over many time points (q datapoints in our case). We added an explanation in the text to clarify this design (pag. 13).

** Normalized root mean of square error, as presented in the paper, is not symmetric, as the denominator only depends on one side. It is a strange property for a distance metric. Again, either clarify this choice or use a symmetric normalisation.*

Thank you for catching this. The denominator in this case refers to the minimum and maximum of either variable - for our case, they are the same because the total population is constant. We fixed the notation and clarified (pag. 12

of the manuscript).

** Since there are two distance metrics (Pearson and NRMSE) I would expect a discussion of their comparison. This should include their theoretical properties, as well as how they relate to the fitting process, and perhaps a discussion of their differences across the outcomes.*

We described the properties of the metrics under the motivation for their selection, with a clarification under the fitting to real-world data section (answered in the previous bullet points, and can be found in the text at page 12). To the Discussion section (pag. 20, at the end of the Effect of the number of parameters subsection), we added limitations and implications for their use in this work. We do not compare the metrics since we use them as a combined assessment - we clarified this in the Methods as we responded to the comments of this list.

– It is not clear what metrics the fitting process relies on. I think it needs some clarification.

We now revised and fixed the typo in Eq. 4n (pag 13), and together with the clarification regarding the metrics, we hope this is clarified. Generally, there are many metrics that can be used in model fitting to real world data; for simplicity and to avoid overwhelming the reader, we utilized the same metrics as for the inter-model comparison. To calculate the two metrics (Pearson's coefficient and NRMSE), we frame the optimization function objectives according to the size of the real-world dataset (clarified in text), and introduce the time factor parameter to adapt their sampling times to the one of the real-world data.

– The paper's conclusion about the preference of ABMs is too optimistic in my opinion. Since the ABMs have more parameters, they are expected to fit real-world data better, which means that this in itself does not evidence the explanatory power of the model. I think an open discussion of the modest conclusions such experiments can support would not reduce the value of the research but rather strengthen it.

We have now included a specific section in the Discussion (pag. 20, subsection Effect of the number of parameters) where we address this issue, particularly to justify our statement. The main idea is that the improved fit results from the model's ability to better represent the underlying real-world dynamics, rather than simply from having more parameters—which, in the case of ABMs, does not necessarily lead to better fitness outcomes.

– The result csv files of the model comparison have been uploaded to git, but I was unable to find the actual piece of code that generates these files. Compiling the whole case study into a one-liner that makes the flow of the work easy to follow would be very useful to enable the transparent presentation of the findings and would help future researchers rely on the results. Making everything executable by a docker file or something similar would be very useful for easy reproducability.

We have updated the repository, which now includes the scripts required to create a docker image that runs the experiments for the two cases. The entry point of the docker image is the script that runs the experiments so once a container is created, it will run all the experiments automatically and will store the results in a docker volume.

More importantly, I did not find the data on which the parameters were fitted.

The data source is here: https://covidsurvey.mit.edu/ and for the analysis in this paper, the selection can also be accessed directly via https://www.nature.com/articles/s41562-022-01347-1#data-availability—a, a source that is cited in the Methods section where the data fitting is presented. We agree that this was not immediately clear, and so we have now revised the text to improve clarity (pag. 13, subsection Infodemic real-world dataset).

With the above changes the paper will be a solid contribution to the journal.

Thank you for your encouraging words. We appreciate your feedback and hope the revisions strengthen the paper's contribution.
