# Supplementary material for: Understanding the mechanisms of infodemics: Equation-based vs. agent-based models
Source: PLoS One. 2025 Dec 17;20(12):e0338614. doi: 10.1371/journal.pone.0338614 (PMC12711016; doi:10.1371/journal.pone.0338614)
Supplement: Attachment — Submitted filename: response to reviewers.docx [file pone.0338614.s009.docx]

*Dear editors and reviewers,*

*Thank you for the opportunity to revise our manuscript, and for the thorough comments. We have revised the paper in light of these useful suggestions. Our responses and changes to the manuscript can be found below (point-by-point for each item) and are as well highlighted in the uploaded revised manuscript.*

**Reviewer #1**

All comments have been addressed and the manuscript has been improved. I believe can now be published

*Answer: Thank you for taking the time to review our manuscript. Your feedback has substantially improved our work.*

**Reviewer #3**

In this paper, the authors present a comparative study of equation-based models (EBMs) and agent-based models (ABMs) to investigate the diffusion mechanisms of misinformation and disinformation, particularly in the context of infodemics during global crises such as the COVID-19 pandemic. The study is grounded in six classical SEIRS-class models and introduces both a simple ABM and an enhanced ABM that incorporates psycho-social dynamics and communication networks. The authors conduct an extensive set of simulations (11,110 experiments) and further validate their models using real-world vaccine acceptance data over 36 weeks. The work is timely, highly relevant, and well-written. However, to be considered for publication, the authors are encouraged to address the following comments, which aim to enhance the overall quality and clarity of the paper.

*Answer: Thank you for this thorough review and the appreciation of our manuscript. Your feedback has substantially improved our work.*

Abstract:

Your abstract should be more explicit to encourage readers to engage with the entire paper. Please consider the following suggestions:

1- The first sentence is somewhat generic; you could make it more compelling by highlighting the impact of false information or by referencing a specific example. For example: “In an era where digital communication accelerates the global spread of false narratives, understanding how misinformation and disinformation propagate, especially during crises such as the COVID-19 pandemic, is vital to public health and policy.”
2- Make the contribution stand out more explicitly by showing what’s new here? For example: “We introduce a novel enhanced ABM that integrates psycho-social factors and communication networks, which are elements often overlooked in traditional EBM frameworks.”
3- The final sentence is informative but could be more assertive in emphasizing the implications. For example: “These findings underscore the critical role of model structure in capturing infodemic dynamics, and advocate for the use of ABMs when psycho-social influences and network interactions are central to the phenomenon.”

*Answer: Thank you for these suggestions, they make the abstract more explicit. We integrated the suggestions and revised the abstract.*

Methods

1. Can you include this link (https://github.com/cristi92b/infodemic_models_comparison) as a footnote with a number instead of putting it explicitly in the text? See also line 566.

*Answer: According to the journal guidelines: "Footnotes are not permitted. If your manuscript contains footnotes, move the information into the main text or the reference list, depending on the content." We thus created a bibliographical entry for the repository.*

2. Each equation should end with either a comma or a full stop. When presenting multiple equations, use commas after each one except the last, which should end with a full stop. The following paragraph must then begin with a capital letter. For example, Equation (1), spanning lines 1–3 of the ODE, should end with a comma; line 4 should end with a full stop, and the word “where” should begin with a capital letter: “Where.” Please review the remaining equations and apply the same formatting consistently.

5. Each equation should be preceded by a specific reference number. For instance, line 239 you should put à text like « ... as shown in Eq. (1) ».

*Answer: We revised both the style of formatting equations and their referencing in text. Thank you for catching this.*

3. Your methodology could be strengthened by including a brief description of the agentbased model (ABM) using the ODD (Overview, Design Concepts, and Details) Protocol proposed by Grimm et al. (https://www.jasss.org/23/2/7.html). This protocol offers a comprehensive framework for clearly communicating ABMs. It facilitates understanding and reproducibility of the model’s design and implementation, and helps researchers, practitioners, and non-experts engage with the work more effectively. Incorporating the ODD protocol will help novice readers better grasp your methodology and findings. For the Simple ABM and the Enhancing ABM, you can:

a. Overview: Describe the purpose, scope, and basic functionality of the model (…).
b. Design Concepts: Include key elements such as agents, agent behavior, environment, and the info spread process (…)
c. Details: Provide information on parameters, initialization, and the simulation process (…)

*Answer: Thank you for this suggestion. We revised the relevant section based on the ODD Protocol.*

4. I appreciate Table 1, but several important criteria are missing that would improve the clarity of the comparison and better highlight the features of the enhanced ABM. I recommend reviewing Table 5 in the paper https://doi.org/10.48550/arXiv.2411.04297 for reference.

*Answer: We combined and extended Table 1 with the characteristics in the example paper, thank you for this suggestion. (Only the table captions is highlighted, but entire table is replaced.)*

Finally, please scan the manuscript again for any remaining typos errors.

*Answer: We performed another proofreading pass (we used US spelling for this manuscript), and hopefully we managed to catch all remaining typos (not highlighted).*

I hope these suggestions are helpful, and I look forward to seeing the revised version.

*Answer: Thank you again for the helpful suggestions!*
